# Supplementary material for: Genetic Diversity of Genes Controlling Unilateral Incompatibility in Japanese Cultivars of Chinese Cabbage
Source: Plants (Basel). 2021 Nov 15;10(11):2467. doi: 10.3390/plants10112467 (PMC8619800; doi:10.3390/plants10112467)
Supplement: Supplementary file 1 [file plants-10-02467-s001.zip › Supplementary files_revise/Figure S1_revise.pdf]

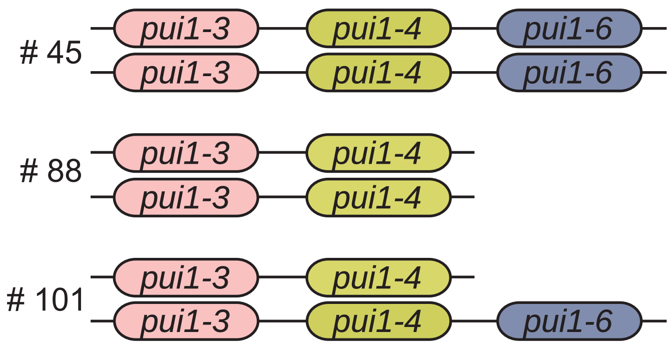

Figure S1. Schematic model of duplicated and triplicated *PUI1* allele.

The genes are close to each other, but the order of genes and physical distances are still unknown.
