# Supplementary material for: Genetic Diversity of Genes Controlling Unilateral Incompatibility in Japanese Cultivars of Chinese Cabbage
Source: Plants (Basel). 2021 Nov 15;10(11):2467. doi: 10.3390/plants10112467 (PMC8619800; doi:10.3390/plants10112467)
Supplement: Supplementary file 1 [file plants-10-02467-s001.zip › Supplementary files_revise/Figure S2_revise.pdf]

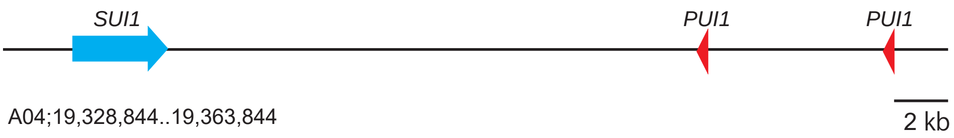

Figure S2. Genomic organization of the *SUI1* and *PUI1* region of *B. rapa* Z1 (*yellow sarson*) identified from published genome sequence available in https://brassicadb.cn [31]. The arrow and arrowheads indicate the direction of transcription of each gene. The region corresponding to the genomic position 19,328,844 to 19,363,844 of A04 chromosome.
