# Supplementary material for: Genetic Diversity of Genes Controlling Unilateral Incompatibility in Japanese Cultivars of Chinese Cabbage
Source: Plants (Basel). 2021 Nov 15;10(11):2467. doi: 10.3390/plants10112467 (PMC8619800; doi:10.3390/plants10112467)
Supplement: Supplementary file 1 [file plants-10-02467-s001.zip › Supplementary files_revise/Figure S3_revise.pdf]

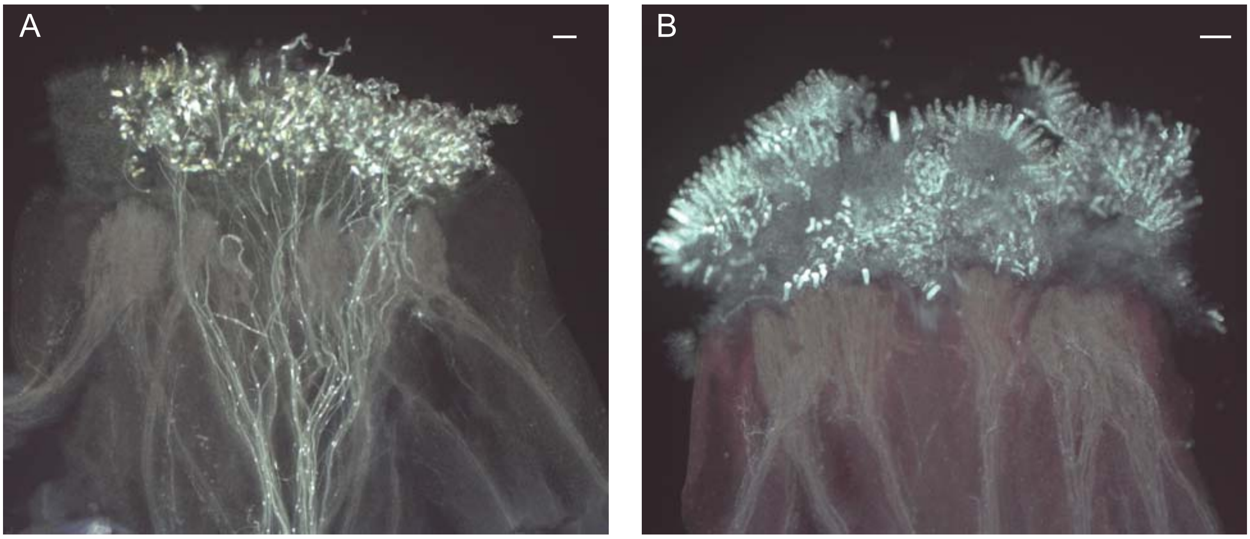

Figure S3. Representative results of test-pollination under UV fluorescence microscopy. (A) Compatible pollination of the stigma from #83 'Taibyou apolo 60' (*SUI1-2/SUI1-10*) with the pollen from $S^{24}t$ (*PUI1-1/PUI1-1*). (B) Incompatible pollination of the stigma from #1 'Mainoumi' (*SUI1-2/SUI1-11*) with the pollen from $S^{24}t$. Scale bars, 100μm.
