# Supplementary material for: Genetic Diversity of Genes Controlling Unilateral Incompatibility in Japanese Cultivars of Chinese Cabbage
Source: Plants (Basel). 2021 Nov 15;10(11):2467. doi: 10.3390/plants10112467 (PMC8619800; doi:10.3390/plants10112467)
Supplement: Supplementary file 1 [file plants-10-02467-s001.zip › Supplementary files_revise/Figure S4_revise.pdf]

```
                            PCP-like1-F1 1st exon
PUI1-1    1:CACCTCTAGTTCATTATCTAAGATGAAAAATTCTTTTCAACCTTCGATTATTGGTTTCTT 60
pui1-3    1:............................................................ 60
pui1-4    1:............................................................ 60
pui1-6    1:............................................................ 60

PUI1-1   61:TATGgtcacagttcttctgttcggttggtatttatatttacaattttattattatattca 120
pui1-3   61:............................................................ 120
pui1-4   61:............................................................ 120
pui1-6   61:............................................................ 120

PUI1-1  121:aaaattatttaaaattaatgttattctgatatcatattattttatgtactgatataattt 180
pui1-3  121:............................................................ 180
pui1-4  121:............................................................ 180
pui1-6  121:............................................................ 180

PUI1-1  181:ttttatcgaattctataattttgggtttagaaattttttccagaataatcaatgattaag 240
pui1-3  181:............................................................ 240
pui1-4  181:.................................a.......................... 240
pui1-6  181:............................................................ 240

PUI1-1  241:aaagaagatgttcatactttaagttttttttccttataaatatgcaaaaaa-ctctctaa 299
pui1-3  241:...................................................a........ 300
pui1-4  241:...................................................-........ 299
pui1-6  241:...................................................-........ 299

PUI1-1  300:gcatatgttttaccaaaaacattaatttctatttaaatacgaaaactaaagttttgtttt 359
pui1-3  301:............................................................ 360
pui1-4  300:............................................................ 359
pui1-6  300:............................................................ 359

                                2nd exon     PUI1-3.4.6F
PUI1-1  360:aactattataataacagGAGTAATGACGACTGCTCAAGCTCAAGTACACAGATTTCCTTG 419
pui1-3  361:............................................G............... 420
pui1-4  360:............................................G............... 419
pui1-6  360:............................................G............... 419

PUI1-1  420:TCGACACATATACAGACCGAAAAATGGAAAATGTGATTTGACCATTTGTTCAGCTGAATG 479
pui1-3  421:.GA...............................A..AC..................... 480
pui1-4  420:.....Sall................................................... 479
pui1-6  420:.GA...............................A..AC..................... 479

PUI1-1  480:TACCAAAGAAAGGGGAAGGCTTGGGATTGGTCGTTGTATGGATCCGCAAAATGAGATGTG 539
pui1-3  481:.................................BamHI...................... 540
pui1-4  480:.....................................G...G.......G.......... 539
pui1-6  480:...................BsrI..C..........GC..G.................. 539
                                   PUI1-3.4.6R
PUI1-1  540:CGCATGCATGCATTATCAAAGGCATAGAAAGAATGAGTGTTTATTTTGATTTTTTTTATC 599
pui1-3  541:............................................................ 600
pui1-4  540:............................................................ 599
pui1-6  540:............................................................ 599

            PCP-like1-R1
PUI1-1  600:TACATTGGAACTCAAATAA 618
pui1-3  601:................... 619
pui1-4  600:................... 618
pui1-6  600:................... 618
```

Figure S4. Nucleotide sequence alignment of *PUI1* alleles. Red lines indicate exon regions. The intron sequences are written in lowercase. Black boxes indicate the primer region used in this study. Red boxes indicate the recognition sites for BamHI, SalI, and BsrI.
