# Supplementary material for: Genetic Diversity of Genes Controlling Unilateral Incompatibility in Japanese Cultivars of Chinese Cabbage
Source: Plants (Basel). 2021 Nov 15;10(11):2467. doi: 10.3390/plants10112467 (PMC8619800; doi:10.3390/plants10112467)
Supplement: Supplementary file 1 [file plants-10-02467-s001.zip › Supplementary files_revise/TableS1.pdf]

Table S1. *PUI1* genotype in the selfed progeny of #101, 'Nanzan'.

Sample number	*PUI1* genotype
#101-1	*pui1-3/pui1-4*
#101-3	*pui1-3/pui1-4*
#101-4	*pui1-3/pui1-4*
#101-5	*pui1-3/pui1-4/pui1-6*
#101-7	*pui1-3/pui1-4/pui1-6*
#101-8	*pui1-3/pui1-4/pui1-6*
#101-9	*pui1-3/pui1-4/pui1-6*
#101-10	*pui1-3/pui1-4/pui1-6*
#101-11	*pui1-3/pui1-4/pui1-6*
#101-12	*pui1-3/pui1-4*
#101-13	*pui1-3/pui1-4/pui1-6*
#101-14	*pui1-3/pui1-4/pui1-6*
#101-15	*pui1-3/pui1-4*
#101-16	*pui1-3/pui1-4/pui1-6*
#101-17	*pui1-3/pui1-4/pui1-6*
#101-18	*pui1-3/pui1-4*
#101-19	*pui1-3/pui1-4/pui1-6*
#101-20	*pui1-3/pui1-4/pui1-6*
#101-21	*pui1-3/pui1-4/pui1-6*
#101-22	*pui1-3/pui1-4/pui1-6*
#101-23	*pui1-3/pui1-4/pui1-6*
#101-24	*pui1-3/pui1-4/pui1-6*
#101-25	*pui1-3/pui1-4*
#101-26	*pui1-3/pui1-4/pui1-6*
#101-27	*pui1-3/pui1-4/pui1-6*
#101-28	*pui1-3/pui1-4/pui1-6*
#101-29	*pui1-3/pui1-4*
#101-30	*pui1-3/pui1-4/pui1-6*
#101-31	*pui1-3/pui1-4*
#101-32	*pui1-3/pui1-4/pui1-6*
#101-33	*pui1-3/pui1-4/pui1-6*
#101-34	*pui1-3/pui1-4/pui1-6*
#101-35	*pui1-3/pui1-4/pui1-6*
#101-36	*pui1-3/pui1-4*
#101-37	*pui1-3/pui1-4/pui1-6*
#101-38	*pui1-3/pui1-4/pui1-6*
#101-39	*pui1-3/pui1-4/pui1-6*
#101-40	*pui1-3/pui1-4/pui1-6*
#101-41	*pui1-3/pui1-4*

#101-42	*pui1-3/pui1-4/pui1-6*
#101-43	*pui1-3/pui1-4/pui1-6*
#101-44	*pui1-3/pui1-4/pui1-6*
#101-45	*pui1-3/pui1-4/pui1-6*
#101-46	*pui1-3/pui1-4/pui1-6*
#101-47	*pui1-3/pui1-4/pui1-6*
#101-48	*pui1-3/pui1-4/pui1-6*
#101-49	*pui1-3/pui1-4*
#101-50	*pui1-3/pui1-4/pui1-6*
#101-51	*pui1-3/pui1-4/pui1-6*
#101-52	*pui1-3/pui1-4/pui1-6*
#101-53	*pui1-3/pui1-4/pui1-6*
#101-54	*pui1-3/pui1-4/pui1-6*
#101-55	*pui1-3/pui1-4/pui1-6*
#101-56	*pui1-3/pui1-4*
#101-57	*pui1-3/pui1-4/pui1-6*
#101-58	*pui1-3/pui1-4/pui1-6*
#101-59	*pui1-3/pui1-4/pui1-6*
#101-60	*pui1-3/pui1-4/pui1-6*
#101-61	*pui1-3/pui1-4*
#101-62	*pui1-3/pui1-4/pui1-6*
#101-63	*pui1-3/pui1-4/pui1-6*
#101-64	*pui1-3/pui1-4/pui1-6*
#101-66	*pui1-3/pui1-4/pui1-6*
#101-67	*pui1-3/pui1-4/pui1-6*
#101-68	*pui1-3/pui1-4/pui1-6*
#101-69	*pui1-3/pui1-4/pui1-6*
#101-70	*pui1-3/pui1-4*
#101-71	*pui1-3/pui1-4/pui1-6*
#101-73	*pui1-3/pui1-4*
#101-74	*pui1-3/pui1-4/pui1-6*
#101-75	*pui1-3/pui1-4/pui1-6*
#101-76	*pui1-3/pui1-4/pui1-6*
#101-77	*pui1-3/pui1-4/pui1-6*
#101-78	*pui1-3/pui1-4/pui1-6*
#101-79	*pui1-3/pui1-4/pui1-6*
#101-80	*pui1-3/pui1-4*
#101-81	*pui1-3/pui1-4/pui1-6*
#101-82	*pui1-3/pui1-4/pui1-6*
