# Supplementary material for: Genetic Diversity of Genes Controlling Unilateral Incompatibility in Japanese Cultivars of Chinese Cabbage
Source: Plants (Basel). 2021 Nov 15;10(11):2467. doi: 10.3390/plants10112467 (PMC8619800; doi:10.3390/plants10112467)
Supplement: Supplementary file 1 [file plants-10-02467-s001.zip › Supplementary files_revise/TableS2.pdf]

Table S2. *SUI1* genotype and stigma-side UI phenotype of selfed progeny of #83.

Population	Sample number	*SUI1* genotype	Stigma-side UI phenotype
#83-S_1	#83-self1	*SUI1-2/SUI1-10*	UC
	#83-self2	*SUI1-2/SUI1-2*	UI
	#83-self4	*SUI1-2/SUI1-10*	UC
	#83-self5	*SUI1-2/SUI1-10*	UC
	#83-self6	*SUI1-10/SUI1-10*	UC
	#83-self8	*SUI1-2/SUI1-10*	UC
	#83-self9	*SUI1-2/SUI1-2*	UI
	#83-self11	*SUI1-10/SUI1-10*	UC
	#83-self12	*SUI1-2/SUI1-10*	UC
	#83-self13	*SUI1-2/SUI1-2*	UI
	#83-self14	*SUI1-10/SUI1-10*	UC
#83-S_2	#83-4self1	*SUI1-2/SUI1-10*	UC
	#83-4self2	*SUI1-2/SUI1-2*	UI
	#83-4self3	*SUI1-2/SUI1-2*	UI
	#83-4self4	*SUI1-2/SUI1-2*	UI
	#83-4self5	*SUI1-10/SUI1-10*	UC
	#83-4self6	*SUI1-10/SUI1-10*	UC
	#83-4self7	*SUI1-2/SUI1-10*	UC
	#83-4self8	*SUI1-2/SUI1-10*	UC
	#83-4self9	*SUI1-2/SUI1-10*	UC
	#83-4self10	*SUI1-2/SUI1-2*	UI
	#83-4self11	*SUI1-2/SUI1-10*	UC
	#83-4self12	*SUI1-2/SUI1-10*	UC
	#83-4self13	*SUI1-2/SUI1-2*	UI
	#83-4self14	*SUI1-10/SUI1-10*	UC
	#83-4self15	*SUI1-2/SUI1-2*	UI
	#83-4self16	*SUI1-2/SUI1-10*	UC
	#83-4self17	*SUI1-2/SUI1-10*	UC
	#83-4self18	*SUI1-10/SUI1-10*	UC
	#83-4self19	*SUI1-2/SUI1-2*	UI
	#83-4self20	*SUI1-2/SUI1-2*	UI
	#83-4self21	*SUI1-2/SUI1-2*	UI
	#83-4self22	*SUI1-2/SUI1-2*	UI
	#83-4self23	*SUI1-10/SUI1-10*	UC
	#83-4self24	*SUI1-10/SUI1-10*	UC
	#83-5self1	*SUI1-10/SUI1-10*	UC

#83-5self2	*SUI1-2/SUI1-10*	UC
#83-5self3	*SUI1-2/SUI1-10*	UC
#83-5self4	*SUI1-2/SUI1-10*	UC
#83-5self5	*SUI1-2/SUI1-2*	UI
#83-5self6	*SUI1-2/SUI1-10*	UC
#83-5self7	*SUI1-2/SUI1-10*	UC
#83-5self8	*SUI1-2/SUI1-10*	UC
#83-5self9	*SUI1-2/SUI1-2*	UI
#83-5self10	*SUI1-2/SUI1-2*	UI
#83-5self11	*SUI1-2/SUI1-10*	UC
#83-5self12	*SUI1-2/SUI1-2*	UI
#83-5self13	*SUI1-2/SUI1-10*	UC
#83-5self14	*SUI1-2/SUI1-10*	UC
#83-5self16	*SUI1-2/SUI1-2*	UI
#83-5self17	*SUI1-2/SUI1-2*	UI
#83-5self18	*SUI1-10/SUI1-10*	UC
#83-5self19	*SUI1-10/SUI1-10*	UC
#83-5self20	*SUI1-2/SUI1-2*	UI
#83-5self21	*SUI1-2/SUI1-10*	UC
#83-5self23	*SUI1-2/SUI1-10*	UC
#83-8self1	*SUI1-2/SUI1-10*	UC
#83-8self2	*SUI1-2/SUI1-2*	UI
#83-8self3	*SUI1-2/SUI1-10*	UC
#83-8self4	*SUI1-2/SUI1-2*	UI
#83-8self5	*SUI1-2/SUI1-2*	UI
#83-8self6	*SUI1-2/SUI1-10*	UC
#83-8self7	*SUI1-2/SUI1-10*	UC
#83-8self8	*SUI1-2/SUI1-10*	UC
#83-8self9	*SUI1-2/SUI1-10*	UC
#83-8self10	*SUI1-2/SUI1-10*	UC
#83-8self11	*SUI1-2/SUI1-2*	UI
#83-8self12	*SUI1-2/SUI1-10*	UC
#83-8self13	*SUI1-2/SUI1-10*	UC
#83-8self14	*SUI1-2/SUI1-10*	UC
#83-8self15	*SUI1-2/SUI1-10*	UC
#83-8self16	*SUI1-2/SUI1-10*	UC
#83-8self17	*SUI1-2/SUI1-10*	UC
#83-8self18	*SUI1-10/SUI1-10*	UC
#83-8self19	*SUI1-2/SUI1-10*	UC

#83-8self20	*SUI1-2/SUI1-10*	UC
#83-8self21	*SUI1-2/SUI1-10*	UC
#83-8self22	*SUI1-2/SUI1-10*	UC
#83-8self23	*SUI1-2/SUI1-10*	UC
#83-8self24	*SUI1-10/SUI1-10*	UC
#83-12self1	*SUI1-2/SUI1-2*	UI
#83-12self3	*SUI1-10/SUI1-10*	UC
#83-12self4	*SUI1-2/SUI1-10*	UC
#83-12self5	*SUI1-10/SUI1-10*	UC
#83-12self6	*SUI1-10/SUI1-10*	UC
#83-12self7	*SUI1-10/SUI1-10*	UC
#83-12self8	*SUI1-2/SUI1-10*	UC
#83-12self9	*SUI1-2/SUI1-10*	UC
#83-12self10	*SUI1-2/SUI1-10*	UC
#83-12self11	*SUI1-10/SUI1-10*	UC
#83-12self12	*SUI1-2/SUI1-2*	UI
