# Supplementary material for: Genetic Diversity of Genes Controlling Unilateral Incompatibility in Japanese Cultivars of Chinese Cabbage
Source: Plants (Basel). 2021 Nov 15;10(11):2467. doi: 10.3390/plants10112467 (PMC8619800; doi:10.3390/plants10112467)
Supplement: Supplementary file 1 [file plants-10-02467-s001.zip › Supplementary files_revise/TableS3.pdf]

Table S3. Primers used in this study.

Name	Sequence
SUI1cDNA_F3	5'-GTGATATGTAGCTTGCATGTGCTAGG-3'
SUI1_gR2	5'-AGTAATGGCAGAGTCAATGTTGGA-3'
PCP-like1-F1	5'-CACCTCTAGTTCATTATCTAAG-3'
PCP-like1-R1	5'-TTATTTGAGTTCCAATGTAG-3'
SUIinter_cF1	5'-CATTCTTGTGGCAGAGTTTCG-3'
SUIinter_cF2	5'-TAACTGTATCAGAGGCTTCGTACCA-3'
SUIinter_cF3	5'-AAGTTCAATTGAACGAGATGGTG-3'
SUIinter_GF1	5'-TATCAAGTAAGAAGTCAATAAGAG-3'
SUI1inter_cF4	5'-CTTGGCTGTTGCGTTCAAGCCAACG-3'
SUIinter_cF5	5'-GAAGCTAACACGAGGAAGGTGGTC-3'
SUI1_2-10typeSDF	5'-GATATGCAAAGAGAGGTGTCTTAGCG-3'
SUI1_2-10typeSDR	5'-ACAAAGAGAGCATAACCGCCTTCG-3'
PUI1-3.4.6-F	5'-GAGTAATGACGACTGCTCAAGCTC-3'
PUI1-3.4.6-R	5'-AAATAAACACTCATCTTTGCTATGCC-3'
