# Supplementary material for: Genetic Diversity of Genes Controlling Unilateral Incompatibility in Japanese Cultivars of Chinese Cabbage
Source: Plants (Basel). 2021 Nov 15;10(11):2467. doi: 10.3390/plants10112467 (PMC8619800; doi:10.3390/plants10112467)
Supplement: Supplementary file 1 [file plants-10-02467-s001.zip › Supplementary files_revise/TableS4.pdf]

Table S4. Accession number of SUI1 and SRK sequences used in phylogenetic analysis.

Name	Accession
SRK^{9}	D30049
SRK^{8}	D38563
SRK^{12}	D38564
SRK^{29}	AB008191
SRK^{40}	AB211197
SRK^{44}	AB201307
SUI1-1	LC088707
SUI1-2	LC088708
SUI1-3	LC088709
SUI1-4	LC088710
SUI1-5	LC088711
SUI1-6	LC088712
SUI1-7	LC088713
SUI1-8	LC088714
SUI1-9	LC088715
SUI1-10	LC641787
SUI1-11	LC641786
SUI1-12	LC641785
